# Dr. J. A. Lewis

**Published:** 1911-09

**Authors:** 


Dr. J. A. Lewis died on August 3 at his home in Lockport, N. Y., after a long illness. Dr. Lewis began practice in Lockport five years ago after many years residence in West Seneca. Dr. Lewis was Health Commissioner of Lockport for one term and at the time of his death was President of the Lockport Tuberculosis Society. He was 70 years of age.

